# Associations of vascular and bone status in arthritis patients

**DOI:** 10.1038/s41598-021-99071-9

**Published:** 2021-09-30

**Authors:** Anita Pusztai, Attila Hamar, Monika Czókolyová, Katalin Gulyás, Ágnes Horváth, Edit Végh, Zsófia Pethő, Szilvia Szamosi, Emese Balogh, Nóra Bodnár, Levente Bodoki, Ágnes Szentpétery, Harjit Pal Bhattoa, György Kerekes, Balázs Juhász, Éva Szekanecz, Katalin Hodosi, Andrea Domján, Sándor Szántó, Hennie G. Raterman, Willem F. Lems, Zoltán Szekanecz, Gabriella Szűcs

**Affiliations:** 1 Division of Rheumatology, Faculty of Medicine, University of Debrecen, Nagyerdei Str 98, Debrecen, 4032 Hungary; 2 Department of Laboratory Medicine, Faculty of Medicine, University of Debrecen, Debrecen, Hungary; 3 Department of Internal Medicine, Faculty of Medicine, University of Debrecen, Debrecen, Hungary; 4 Department of Sports Medicine, Faculty of Medicine, University of Debrecen, Debrecen, Hungary; 5 Department of Oncology, Faculty of Medicine, University of Debrecen, Debrecen, Hungary; 6 Centre for Arthritis and Rheumatic Diseases, St. Vincent’s University Hospital, Dublin, Ireland; 7 Department of Rheumatology, Uppsala University Hospital, Uppsala, Sweden; 8 Department of Rheumatology, Northwest Clinics, Alkmaar, The Netherlands; 9 Amsterdam Rheumatology and Immunology Centre, Amsterdam, The Netherlands

**Keywords:** Immunology, Diseases, Rheumatology

## Abstract

Cardiovascular (CV) disease and osteoporosis (OP) have been associated with rheumatoid arthritis (RA) and ankylosing spondylitis (AS). Bone and vascular biomarkers and parameters along with the effect of 1-year anti-TNF therapy on these markers were assessed in order to determine correlations between vascular pathophysiology and bone metabolism in RA and AS. Thirty-six patients treated with etanercept or certolizumab pegol and 17 AS patients treated with ETN were included in a 12-month follow-up study. Bone and vascular markers were previously assessed by ELISA. Bone density was measured by DXA and quantitative CT (QCT). Flow-mediated vasodilation (FMD), common carotid intima-media thickness (IMT) and pulse-wave velocity (PWV) were assessed by ultrasound. Multiple correlation analyses indicated associations between bone and vascular markers. Osteoprotegerin, sclerostin and cathepsin K were significantly associated with FMD, IMT and PWV, respectively (p < 0.05). Moreover, total and trabecular BMD determined by QCT inversely correlated with IMT (p < 0.05). On the other hand, among vascular parameters, platelet-derived growth factor BB and IMT correlated with DXA femoral and QCT total BMD, respectively (p < 0.05). In the RM-ANOVA analysis, anti-TNF treatment together with baseline osteocalcin, procollagen 1 N-terminal propeptide (P1NP) or vitamin D3 levels determined one-year changes in IMT (p < 0.05). In the MANOVA analysis, baseline disease activity indices (DAS28, BASDAI), the one-year changes in these indices, as well as CRP exerted effects on multiple correlations between bone and vascular markers (p < 0.05). As the pattern of interactions between bone and vascular biomarkers differed between baseline and after 12 months, anti-TNF therapy influenced these associations. We found a great number of correlations in our RA and AS patients undergoing anti-TNF therapy. Some of the bone markers have been associated with vascular pathophysiology, while some vascular markers correlated with bone status. In arthritis, systemic inflammation and disease activity may drive both vascular and bone disease.

## Introduction

Cardiovascular (CV) disease (CVD) and osteoporosis (OP) are crucial health problems^1,2^. CVD and OP may occur simultaneously in the general population. Furthermore, both CVD and OP have also been associated with inflammatory rheumatic musculoskeletal diseases, such as rheumatoid arthritis (RA) and ankylosing spondylitis (AS)^1,3–7^. There are common pathogenic pathways in inflammatory atherosclerosis and bone loss^1,3,7–10^. Targeted therapies that are used to treat RA and AS may improve CVD and OP secondary to these rheumatic diseases^1,3,4,8,11^.

Relatively few studies assessed associations between CVD and OP or fragility fractures in these patients. The risks of both CVD and fractures are roughly doubled in RA^1,3–5,12,13^. In one recent study, in RA patients with fractures, there was a 1.8-times increased risk for CVD^14^. In the Oslo RA register, CV death was associated with increased prevalence of OP and previous fractures^15^.

Both conventional and inflammatory risk factors are involved in the pathogenesis of arthritis-associated atherosclerosis and OP^1^. Traditional risk factors include age, postmenopausal status and/or oestrogen deprivation, physical inactivity, smoking and diabetes mellitus^1,16–19^. Inflammatory cells, acute phase reactants, several pro-inflammatory cytokines including tumor necrosis factor α (TNF-α), chemokines, angiogenic growth factors, adipokines and other mediators including osteoprotegerin, Receptor Activator of Nuclear Factor κB Ligand (RANKL), sclerostin, vitamin D, parathyroid hormone (PTH) and cathepsin K are associated with both events^1,7,9,17,18,20–25^. Among cytokines, elevated TNF-α levels have been associated with both myocardial infarction and fragility fractures^17,26^.

Surrogate markers of atherosclerosis and bone loss have been investigated in arthritides. Among ultrasound-based techniques, common carotid intima-media thickness (IMT) or coronary artery CT, brachial artery flow-mediated vasodilation (FMD) and arterial pulse-wave velocity (PWV) are suitable to detect overt atherosclerosis, endothelial dysfunction and vascular stiffness, respectively^27^. In non-rheumatic cohorts, higher IMT^28^ or coronary artery calcification scores^29,30^ were associated with lower BMD. In RA patients, there was an inverse correlation between osteoprotegerin levels and FMD^31^.

Targeted therapies, such as TNF-α inhibitors may be beneficial for secondary CVD^4,11,32–35^ and OP^8,35–38^ associated with arthritis. However, these studies separately assessed the vascular or bone effects of anti-TNF drugs including clinical outcomes and/or surrogate markers. In a prospective early RA cohort, the use of biologics was inversely associated with the risk of comorbidity development^39^.

We have not found any studies, where atherosclerosis and bone metabolism were assessed in the very same cohort. We have recently set up a mixed cohort of RA and AS patients and assessed either vascular^40,41^ or bone status^42–44^. The vascular and bone results partially referred to in this paper have been published^40,42^. As now we wished to investigate atherosclerosis and osteoporosis, both driven by systemic inflammation, in parallel, in the same patient cohort. We also compared baseline and post-treatment data in order to assess the effects of biological therapy on vascular-bone associations.

## Patients and methods

Methods have been applied as reported previously^40,42^.

### Patients

Fifty-three patients with RA (n = 36) and AS (n = 17) selected for the initiation of anti-TNF therapy but unselected for CVD and OP were enrolled in the study. Patient characteristics are seen in Table 1. The cohort included 34 women and 19 men with mean age of 52.0 ± 12.1 (range 24–83) years, while mean age at diagnosis was 43.5 ± 12.1 (range 23–62) years. Mean disease duration was 8.5 ± 7.9 (range 1–44) years. Exclusion criteria included untreated, unstable hypertension (blood pressure > 140/90 mmHg), current inflammatory disease other than RA or AS, infectious disease or renal failure (based on eGFR and hospital records). None of the patients had known primary osteoporosis prior to the diagnosis of RA or AS. None of patients received aspirin, clopidogrel, heparin and warfarin at the time of inclusion. Some patients could have taken vitamin D and/or calcium in the past, however, none of them received supplementation at least 3 months prior to baseline. As antihypertensive drugs may affect the vascular status, hypertension had been stabilized for at least 6 months before the onset of this study. Moreover, antihypertensive drugs remained unchanged throughout the study. Patients with active disease were recruited prior to initiating a biological therapy. At baseline RA and AS patients had a mean DAS28 of 5.00 ± 0.86 and a mean BASDAI of 5.79 ± 1.19, respectively. All patients started on an anti-TNF therapy at baseline and continued the same biological treatment during one year. Clinical assessments were performed at baseline, and after 6 and 12 months of therapy. Among the 36 RA patients, 20 received subscutaneous etanercept 50 mg/week and 16 received certolizumab pegol (400 mg at 0, 2 and 4 weeks, and thereafter 200 mg twice weekly). Altogether 18 RA patients were treated with etanercept and 13 with certolizumab pegol in combination with methotrexate. Other patients received anti-TNF monotherapy. RA patients did not take DMARDs other than methotrexate. All 17 AS patients received etanercept monotherapy 50 mg/week. Altogether 12 RA and 2 AS patients currently took low-dose (< 6 mg/day) methylprednisolone (Table 1). The study was approved by the Hungarian Scientific Research Council Ethical Committee (approval No. 14804-2/2011/EKU). Written informed consent was obtained from each patient and assessments were carried out according to the Declaration of Helsinki.

**Table 1:** Patient characteristics.

	RA	AS	Total
n	36	17	53
Female:male	31:5	3:14	34:19
Age (mean ± SD)(range), years	55.9 ± 9.8 (35–83)	43.6 ± 12.4 (24–72)	52.0 ± 12.1 (24–83)
Disease duration (mean ± SD) (range), years	9.1 ± 8.3 (1–44)	7.2 ± 7.0 (1–26)	8.5 ± 7.9 (1–44)
Menopausal status (number of women)	25	1	26
Menopause duration (in menopausal women), years	11.0 ± 7.6	23.0	12.0 ± 7.8
Menopausal hormone replacement (number of women)	7	1	8
BMI (mean ± SD), kg/m^2^	29.3 ± 3.6	31.1 ± 3.8	29.9 ± 3.7
Obesity (BMI > 30 kg/m^2^)	17	11	28
Smoking (current)	7	7	14
Positive CV history	8	1	9
Diabetes mellitus history	3	1	4
Hypertension history	17	4	21
Fragility fracture history (events/patients)	14/10	8/7	22/17
Corticosteroid use at baseline (mean ± SD), years	3.8 ± 5.9	0.6 ± 0.9	2.8 ± 5.1
RF positivity, n (%)	26 (72)	–	–
ACPA positivity, n (%)	21 (58)	–	–
DAS28 (baseline) (mean ± SD)	5.00 ± 0.86	–	–
BASDAI (baseline) (mean ± SD)	–	5.79 ± 1.19	–
Treatment (ETN, CZP)	20 ETN, 16 CZP	17 ETN	37 ETN, 16 CZP

*ACPA*, anti-citrullinated protein antibody, *AS*, ankylosing spondylitis, *BASDAI* Bath ankylosing spondylitis disease activity score, *BMI* body mass index, *CV* cardiovascular, *CZP* certolizumab pegol, *DAS28* 28-joint disease activity score, *ETN* etanercept, *RA* rheumatoid arthritis, *RF* rheumatoid factor, *SD* standard deviation.

### Clinical assessment

First, detailed medical history was taken and body mass index (BMI) was determined (Table 1). Some patients had previous fragility fractures, however, no new fractures occurred during the study. Further clinical assessments including physical examination were performed at baseline, and after 6 and 12 months of therapy.

### Bone densitometry assessments by DXA and QCT

The DXA and QCT assessments carried out in the very same cohort were performed and published previously^42^.

### Assessment of vascular physiology by ultrasound

The FMD, IMT and PWV assessments carried out in the very same cohort were performed and published previously^40^.

### Laboratory measurements and assessment of disease activity

Assessments of C-reactive protein (CRP), IgM rheumatoid factor (RF) and ACPA (anti-CCP) autoantibodies, as well as disease activity assessments (DAS28, BASDAI) were performed and reported previously^40–42^.

### Bone biomarkers

Bone biomarkers including serum calcium, phosphate, parathyroid hormone, 25-OH-vitamin D3, osteocalcin, procollagen type I N-propeptide (P1NP), C-terminal collagen telopeptide (CTX), osteoprotegerin, sclerostin, Dickkopf-1 (DKK-1), soluble RANKL (sRANKL) and cathepsin K were determined in the very same cohort and reported previously^42^.

### Vascular and angiogenic biomarkers

Vascular and angiogenic biomarkers including vascular endothelial growth factor (VEGF), platelet-derived growth factor BB (PDGF-BB), angiopoietin 1, angiopoietin 2, thrombospondin 1 and brain natriuretic peptide (BNP) fragments were determined in the very same cohort and reported previously^41^.

### Statistical analysis

Statistical analysis was performed using SPSS version 22.0 (IBM) software (https://www.ibm.com/analytics/spss-statistics-software) as reported previously^40,42^. Data are expressed as the mean ± SD for continuous variables and percentages for categorical variables. The distribution of continuous variables was evaluated by Kolmogorov–Smirnov test. Independent and paired two-tailed t-test were used to assess the differences. Nominal variables were compared between groups using the chi-squared or Fisher’s exact test, as appropriate. Correlations were determined by Pearson’s or Spearman’s analyses. Univariable and multivariable regression analysis using the stepwise method were applied to investigate independent associations between vascular or bone imaging and biomarkers (dependent variables) and other clinical, laboratory and imaging parameters (independent variables). The β standardized linear coefficients showing linear correlations between two parameters associated with each other. The B (+ 95% CI) regression coefficient indicated independent associations between dependent and independent variables during changes. General linear model (GLM) repeated measures analysis of variance (RM-ANOVA) was performed in order to determine the additional effects of multiple parameters including therapy on 12-month changes of vascular or bone markers. Similarly, GLM multivariate regression analysis (MANOVA) was performed in order to assess covariate determinants of two correlating vascular and bone markers. In both analyses, partial η^2^ is given as indicator of effect size, with values of 0.01 suggesting small, 0.06 medium and 0.14 large effects. The reliability was tested by inter-item correlation and intraclass correlation (ICC). With respect to the FMD, IMT and PWV tests, ICC = 0.470; F-test value: 1.887; p = 0.001. The power was estimated with G*Power software version 3.1.9.7 (https://www.psychologie.hhu.de/arbeitsgruppen/allgemeine-psychologie-und-arbeitspsychologie/gpower). p values < 0.05 were considered significant.

### Ethics approval and consent to participate

The study was approved by the Hungarian Scientific Research Council Ethical Committee (approval No. 14804-2/2011/EKU). Written informed consent was obtained from each patient and assessments were carried out according to the Declaration of Helsinki.

## Results

### Associations between biomarkers of vascular and bone status

Separate vascular^40,41^ and bone^42–44^ imaging and laboratory marker data obtained in this patient cohort have been published. Here we reanalyzed the data and assessed the relationship between bone and vascular status in these patients. None of the data presented here have been published previously.

**Figure 1:**
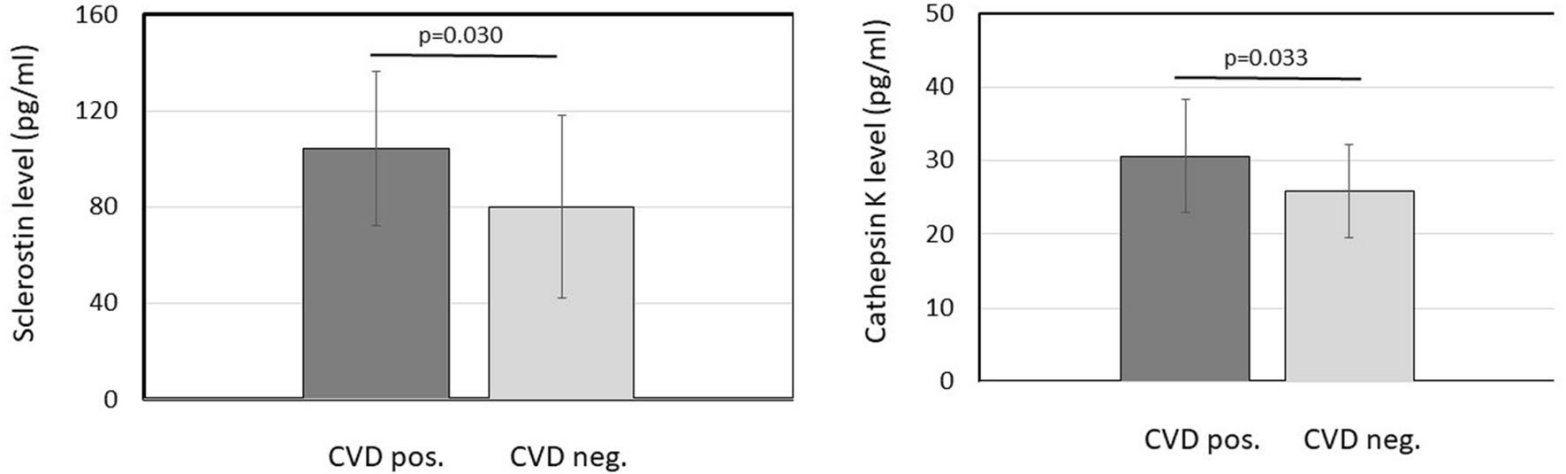
Levels of sclerostin and cathepsin K in arthritis patients with and without cardiovascular (CV) history. Serum concentrations of both primarily bone biomarkers are significantly increased in patients with positive history of CV disease.

Patients with history of CVD had significantly higher baseline sclerostin (104.1 ± 32.1 vs 80.1 ± 37.8 pmol/l; p = 0.030) and cathepsin K levels (30.6 ± 7.7 vs 25.8 ± 6.3 pmol/l; p = 0.033) compared to those with negative CV history (Fig. 1).

Results of statistical analyses described above are included in Tables 2, 3, 4 and 5. Here, we present only associations regarding baseline and 12-month data. When vascular and bone imaging parameters were compared, IMT variably and inversely correlated with total and trabecular BMD as determined by QCT (Table 2). Similarly, PWV-12 inversely correlated with DXA femoral neck BMD, as well as with QCT total BMD (Table 2). Regarding associations between vascular imaging and bone laboratory marker data, baseline FMD and osteoprotegerin positively correlated with each other. IMT positively correlated with sclerostin and inversely with osteocalcin, P1NP and vitamin D3 (Table 2). PDGFBB positively correlated with DXA femoral BMD (Table 2). Various vascular and bone laboratory biomarkers also showed correlations with each other (Table 2).

**Table 2:** Significant correlations between vascular and bone biomarkers.

Vascular biomarker	Bone biomarker	R value	p value
**Vascular imaging vs bone imaging**			
*IMT-0*	*QCTTOTBMD-12*	− 0.314	0.046
*IMT-0*	*QCTTRABBMD-0*	− 0.355	0.023
*IMT-6*	*QCTTOTBMD-0*	− 0.395	0.011
*IMT-6*	*QCTTOTBMD-12*	− 0.387	0.012
*IMT-6*	*QCTTRABBMD-0*	− 0.496	0.001
*IMT-6*	*QCTTRABBMD-12*	− 0.339	0.030
*IMT-12*	*QCTTOTBMD-0*	− 0.437	0.004
*IMT-12*	*QCTTOTBMD-12*	− 0.543	< 0.001
*IMT-12*	*QCTTRABBMD-0*	− 0.366	0.019
*IMT-12*	*QCTTRABBMD-12*	− 0.442	0.004
*PWV-12*	*QCTTOTBMD-12*	− 0.333	0.033
**Vascular imaging vs bone laboratory biomarker**			
*FMD-0*	*OPG-0*	0.343	0.041
*FMD-6*	*OPG-6*	0.460	0.002
*FMD-6*	*OPG-0*	0.503	0.001
*IMT-0*	*SOST-0*	0.346	0.039
*IMT-12*	*SOST-12*	0.347	0.023
*IMT-12*	*OC-12*	− 0.307	0.032
*IMT-12*	*P1NP-12*	− 0.293	0.041
*IMT-12*	*VITD3-0*	− 0.337	0.018
*PWV-6*	*PTH-6*	0.306	0.036
*PWV-12*	*CATHK-0*	0.376	0.013
*PWV-12*	*CATHK-12*	0.352	0.021
**Vascular laboratory biomarker vs bone imaging**			
*PDGFBB-0*	*DEXFEMBMD-0*	0.385	0.043
*PDGFBB-0*	*DEXFEMBMD-12*	0.434	0.021
**Vascular laboratory biomarker vs bone laboratory biomarker**			
*ANG1-0*	*DKK1-0*	0.669	< 0.001
*ANG1-6*	*DKK1-6*	0.513	0.039
*ANG2-0*	*DKK1-0*	0.467	0.018
*ANG2-0*	*OPG-0*	0.486	0.014
*ANG2-6*	*OPG-6*	0.486	0.014
*ANG2-6*	*RANKL-6*	− 0.440	0.028
*ANG2-12*	*OPG-12*	0.698	< 0.001
*PDGFBB-0*	*DKK1-0*	0.545	0.005
*PDGFBB-12*	*DKK1-12*	0.516	0.008
*TSP1-0*	*OPG-0*	0.589	0.002
*TSP1-0*	*SOST-0*	0.466	0.019
*TSP1-0*	*SOST-12*	0.457	0.022
*BNP-0*	*CTX-0*	0.289	0.048
*BNP-12*	*CTX-0*	0.374	0.010
*BNP-12*	*OC-12*	0.321	0.028

*ANG1* angiopoietin 1, *ANG2* angiopoietin 2, *BMD* bone mineral density, *BNP* brain-type natriuretic peptide, *CATHK* cathepsin K, *CTX* C-terminal telopeptide, *DEXFEMBMD* DXA femoral neck BMD, *DKK1* Dickkopf-1, *FMD* flow-mediated vasodilation, *IMT* carotid intima-media thickness, *OC* osteocalcin, *OPG* osteoprotegerin, *PDGFBB* platelet-derived growth factor BB, *P1NP* procollagen type I N-propeptide, *PTH* parathyroid hormone, *PWV* pulse-wave velocity, *QCTTOTBMD* QCT total BMD, *QCTTRABBMD* QCT trabecular BMD; *RANKL* Receptor Activator of Nuclear κB Ligand, *SOST* sclerostin, *TSP1* thrombospondin 1, *VITD3* vitamin D3.

**Table 3:** Univariable and multivariable analysis of determinants of vascular and bone status.

Dependent variable	Independent variable	Univariable analysis				Multivariable analysis			
		β	p	B	95% CI	β	p	B	95% CI
**Determinants of vascular status by bone biomarkers**									
*FMD-0*	*OPG-0*	0.343	0.041	0.620	0.027–1.213	0.356	0.026	0.674	0.088–1.259
*FMD-6*	*OPG-0*	0.503	0.001	1.101	0.488–1.714				
	*OPG-6*	0.460	0.002	1.055	0.396–1.714				
*IMT-0*	*SOST-0*	0.346	0.039	0.001	0–0.002				
	*QCTTRABBMD-0*	− 0.355	0.023	− 0.012	− 0.022–− 0.002	− 0.355	0.023	− 0.012	− 0.022–− 0.002
*IMT-6*	*OC-0*	− 0.400	0.005	− 0.005	− 0.009–− 0.002	− 0.300	0.032	− 0.003	− 0.006–0
	*P1NP-0*	− 0.330	0.023	− 0.002	− 0.004–− 0				
	*QCTTOTBMD-0*	− 0.395	0.011	− 0.026	− 0.046–− 0.006				
	*QCTTRABBMD-0*	− 0.496	0.001	− 0.017	− 0.027–− 0.007	− 0.440	0.002	− 0.015	− 0.025–− 0.006
*IMT-12*	*OC-12*	− 0.307	0.032	− 0.004	− 0.007–0				
	*CTX-12*	− 0.308	0.031	− 0.214	− 0.407–− 0.020				
	*P1NP-12*	− 0.293	0.041	− 0.001	− 0.002–0				
	*VITD3-0*	− 0.337	0.018	− 0.001	− 0.003–0				
	*SOST-12*	0.347	0.023	0.001	0–0.001	0.379	0.014	− 0.001	0–0.002
	*QCTTOTBMD-0*	− 0.437	0.004	− 0.030	− 0.051–− 0.010				
	*QCTTOTBMD-12*	− 0.543	< 0.001	− 0.036	− 0.055–− 0.018	− 0.390	0.011	− 0.024	− 0.041–− 0.006
	*QCTTRABBMD-0*	− 0.366	0.019	− 0.013	− 0.025–− 0.002				
	*QCTTRABBMD-12*	− 0.442	0.004	− 0.015	− 0.025–0.005				
*PWV-0*	*VITD3-0*	-0.316	0.042	0.016	0.001–0.032	− 0.316	0.042	0.016	0.001–0.032
*PWV-6*	*PTH-6*	0.306	0.036	0.310	0.021–0.599	0.275	0.047	0.278	0.004–0.553
*PWV-12*	*CATHK-0*	0.376	0.013	0.103	0.023–0.182	0.376	0.013	0.103	0.023–0.182
	*CATHK-12*	0.352	0.021	0.122	0.020–0.224				
	*DEXFEMBMD-0*	− 0.361	0.014	− 7.017	− 12.532–− 1.501				
	*QCTTOTBMD*-*12*	− 0.333	0.033	− 0.608	− 1.165–− 0.051				
**Determinants of bone status by vascular biomarkers**									
*DEXFEMBMD-0*	*PDGFBB-0*	0.385	0.043	0					
*DEXFEMBMD-12*	*PDGFBB-0*	0.434	0.021	0		0.357	0.036	0	
	*PDGFBB-6*	0.410	0.030	0					
*QCTTOTBMD-12*	*IMT-0*	− 0.314	0.046	− 5.053	− 10.003–− 0.104				
	*IMT-6*	− 0.387	0.012	− 6.114	− 10.834–− 1.394				
	*IMT-12*	− 0.543	< 0.001	− 8.093	− 12.149–− 4.037	− 0.543	< 0.001	− 8.093	− 12.149–− 4.037
	*PWV12*	− 0.333	0.033	− 0.183	− 0.350–− 0.015				
*QCTTRABBMD-0*	*IMT-0*	− 0.355	0.023	− 10.425	− 19.318–− 1.532				
	*IMT-6*	− 0.339	0.030	− 10.430	− 19.817–− 1.043				
	*IMT-12*	− 0.442	0.004	− 12.837	− 21.282–− 4.393				

*BMD* bone mineral density, *CATHK* cathepsin K, *CTX* C-terminal telopeptide, *DEXFEMBMD* DXA femoral neck BMD, *FMD* flow-mediated vasodilation, *IMT* carotid intima-media thickness, *OC* osteocalcin, *OPG* osteoprotegerin, *PDGFBB* platelet-derived growth factor BB, *P1NP* procollagen type I N-propeptide, *PTH* parathyroid hormone, *PWV* pulse-wave velocity, *QCTTOTBMD* QCT total BMD, *QCTTRABBMD* QCT trabecular BMD, *SOST* sclerostin, *VITD3* vitamin D3.

**Table 4:** Significant results of general linear model (GLM) repeated measures analysis of variance (RM-ANOVA) test determining the effects of treatment and other independent variables on IMT as dependent variable.

Dependent variable	Effect	F	p	Partial η^2^
*IMT 0-6-12*	Treatment * OC-0(inv)	3.357	0.045	0.147
*IMT 6-12*		4.598	0.038	0.103
*IMT 0-6-12*	Treatment * P1NP-0(inv)	3.623	0.036	0.157
*IMT 0-6*		5.471	0.024	0.120
*IMT 6-12*	Treatment * VITD3-0(inv)	4.459	0.041	0.100

*GLM* general linear model, *IMT* carotid intima-media thickness, *inv* inverse, *OC* osteocalcin, *P1NP* procollagen type I N-propeptide, *RM-ANOVA* repeated measures analysis of variance, *VITD3* vitamin D3.

**Table 5:** Significant results of general linear model (GLM) multivariate analysis of variance (MANOVA) test determining the effects of an independent variable on the association of two dependent variables.

Dependent variables	Effect	F	p	Partial η^2^
*IMT-12* and *QCTTRABBMD-12 (inv)*	*DAS28-0 *or *BASDAI-0*	3.962	0.028	0.185
*PWV-12 *and *QCTTRABBMD-12 (inv)*	*DAS28-0 *or* BASDAI-0*	3.482	0.042	0.166
*ANG2-6* and* RANKL-6 (inv)*	*DAS28-0 i BASDAI-0*	4.748	0.021	0.322
*OC-12* and* BNP-12 (pos)*	*CRP-0*	3.764	0.031	0.146
*IMT-12* and* SOST-12*	*ΔDAS28*_*0–12*_ or *ΔBASDAI*_*0–12*_	4.271	0.021	0.176
*PWV-12* and* CATHK-12*	*ΔDAS28*_*0–12*_*iΔBASDAI*_*0–12*_	4.243	0.021	0.175

*ANG2* angiopoietin 2, *BASDAI* Bath ankylosing spondylitis disease activity index, *BNP* B-type natriuretic peptide, *CRP* C-reactive protein, *DAS28* 28-joint disease activity score, *GLM* general linear model, *IMT* carotid intima-media thickness, *inv* inverse, *OC* osteocalcin, *MANOVA* multivariate analysis of variance, *pos* positive, *PWV* pulse-wave velocity, *QCTTRABBMD* QCT trabecular bone mineral density, *RANKL* receptor activator nuclear κB.

Both univariable and multivariable analyses of determinants of vascular status confirmed independent associations between osteoprotegerin and FMD, sclerostin and IMT, as well as cathepsin K and PWV. In addition, QCT trabecular BMD was inversely associated with IMT, while QCT total BMD with IMT. Moreover, we found association of vitamin D3 with PWV (Table 3). On the other hand, when determinants of bone status by vascular parameters was analyzed, both multivariable and univariable analyses suggested that PDGFBB positively associated with DXA femoral BMD, while IMT inversely correlated with QCT total BMD (Table 3).

RM-ANOVA was performed in order to assess independent determinants of 12-month changes in vascular or bone markers as dependent variables. Only IMT as dependent variable yielded significant results. In this respect, anti-TNF treatment exerted combined effects with either baseline osteocalcin, P1NP or vitamin D3 (all inverse relationship with IMT) on changes in IMT (Table 4).

MANOVA was performed in order to study independent determinants of two associated vascular and bone markers and dependent variables. Baseline disease activity indices (DAS28 or BASDAI) had significant effects on the inverse associations between IMT and QCT trabecular BMD, as well as on those between PWV and QCT trabecular BMD. Moreover, CRP exerted significant effects on the positive correlation between OC and BNP (Table 5). We also assessed the effects of anti-TNF treatment-related changes (Δ) of disease activity between baseline and 12 months on various biomarkers. ΔDAS28_0–12_ or ΔBASDAI_0–12_ exerted significant effects on the positive associations between IMT and sclerostin, as well as PWV and cathepsin K (Table 5).

Finally, we compared the patterns of bone and vascular marker associations at baseline versus 12 months in order to further determine the complex effects of anti-TNF therapy on inflammatory bone-vascular interactions. Patients at baseline have high disease activity, while their status after 12 months of treatment in most of them represents a rather non-inflammatory state. In the uni- and multivariable analyses, associations between osteoprotegerin and FMD, sclerostin and IMT or PDGFBB and DXA femoral BMD were observed at baseline but not after 12 months (Table 3). On the other hand, correlations between osteocalcin, CTX, P1NP, vitamin D3 and sclerostin with IMT, those between QCT total BMD and PWV, as well as those between QCT total BMD and IMT or PWV were seen only after 12 months (Table 3). Correlations between one marker at baseline with another one after 12 months showed that baseline vascular markers could determine bone status after one year or vice versa (Table 3). In the MANOVA analysis, as described above, baseline CRP, disease activity or changes in disease activity overtime determined several correlations between bone and vascular markers in treated patients after 12 months (Table 5). All these observations underscore that anti-TNF treatment influenced bone-vascular interactions over time.

## Discussion

Previously, we separately studied and reported vascular^40^ and bone status^42^ in the same cohort. Here we wished to study atherosclerosis and osteoporosis, both driven by inflammation, in parallel. Therefore we reanalysed previously obtained vascular and bone data. In addition, we compared pre- and post-treatment data in order to determine the possible effects of TNF inhibition on the relationship between vascular and bone status in RA and AS. Our present study demonstrates that in anti-TNF treated RA and AS patients, vascular and bone parameters show numerous correlations. Some bone markers may predict vascular status after one-year treatment and vice versa. Systemic inflammation and arthritic disease activity may influence the associations between bone and vascular biomarkers.

Both CVD and OP have been associated with inflammatory rheumatic diseases, such as RA and AS. There have been numerous studies on the effects of targeted therapies on CVD, OP and their biomarkers in arthritides^1–12,40–42^. However, very few studies assessed CVD and OP simultaneously in the same patient cohort in an observational follow-up. A population-based cohort study by Ni Mhuircheartaighet et al.^14^ indicated a substantial increase of CVD development in RA patients with fragility fractures. Moreover, 15-year follow-up data from the Oslo RA Register revealed that RA patients, who deceased from CV or cerebrovascular disease, also more often had OP and previous fractures compared to those who did not die of CVD^15^.

There are imaging and laboratory biomarkers of arthritis-associated atherosclerosis and bone loss. Moreover, some of the biomarkers also have pathogenic role in these comorbidities^1,7,9,17,18,20–25^. DXA and QCT are suitable to detect bone density and also some aspects of bone quality^3,45^, while IMT, FMD and PWV detect manifest atherosclerosis, endothelial dysfunction and vascular stiffness, respectively^27^. Molecular markers that link inflammatory atherosclerosis and OP include markers of systemic inflammation including CRP, pro-inflammatory cytokines including TNF-α, angiogenic mediators (PDGF-BB, angiopoietin 1 and 2, thrombospondin 1), BNP and markers of bone metabolism (osteoprotegerin, RANKL, sclerostin, 25-OH-vitamin D3, PTH and cathepsin K)^1,7,9,17,18,20–25^. TNF-α may play a central role in vascular and bone pathology and the development of arthritis-associated CVD and OP^17,26^. Indeed, anti-TNF therapy may control inflammatory atherosclerosis^4,11,32–35^ and bone loss^8,35–38^. There have been few reports on associations between vascular and bone surrogate markers in non-arthritic cohorts^28–30^. However, only Delgado-Frias et al.^31^ assessed such markers in RA and found correlation between FMD and OPG in that cohort. Thus, apart from the very few studies that linked non-inflammatory CVD and OP, imaging and laboratory biomarkers of CVD and OP have not yet been assessed at the same time in anti-TNF-treated inflammatory arthritis patients.

In the same cohort, we have previously analysed the effects of one-year anti-TNF therapy on vascular and bone status and published the results separately (vascular^40,41^; bone^42–44^). Here we found a great number of correlations between vascular and bone surrogate markers. The most relevant ones include significant inverse correlations between IMT and QCT parameters, as well as between PWV and femoral neck BMD at different time points. Thus, not only association between inflammatory biomarkers, which may change rapidly, was observed but structural markers were also related. These data suggest that sustained systemic inflammation may exert chronic, simultaneous effects on the bone and vessels. We also confirmed the positive association between FMD and osteoprotegerin reported by Delgado-Frias et al.^31^. Our findings showing that serum osteoprotegerin levels associated with FMD both prior to and following anti TNF treatment further support its role as a biomarker of vascular risk and prognosis. Moreover, IMT and PWV exerted correlations with numerous bone biomarkers. Among vascular biomarkers, PDGFBB positively correlated with femoral neck BMD and multiple angiogenic markers correlated with numerous bone biomarkers. With regards to the regression assessments, baseline osteoprotegerin may positively associate with baseline FMD and 12-month sclerostin with IMT-12. In addition, QCT trabecular BMD at baseline and total BMD after 12 months inversely correlated with baseline and 12-month IMT, respectively. On the other hand, 12-month IMT also inversely associated with QCT total BMD after 12 months. These results show that IMT may be closely associated with total and trabecular BMD as measured by QCT and provide further evidence that atherosclerosis and inflammatory bone loss may be associated. Finally, baseline vitamin D3 inversely associated with baseline PWV. Higher PWV may rather be associated with lower femoral neck BMD as determined by DXA and with low vitamin D3 levels. FMD did not show any significant associations with DXA or QCT BMD, but rather with osteoprotegerin.

Some biomarkers at baseline also significantly associated with other parameters at later time points. Baseline cathepsin K positively correlated with PWV after 12 months. On the other hand, baseline PDGFBB levels were positive determinants of femoral neck BMD after 12 months. Moreover, as suggested by the RM-ANOVA analysis, one-year biologic treatment combined with baseline levels of different bone biomarkers may predict changes of IMT upon therapy. Finally, MANOVA analysis revealed that systemic inflammation (CRP) or disease activity, as well as their anti-TNF-related changes between baseline and 12 months of treatment may significantly influence the interrelationship between certain bone and vascular biomarkers.

It may also be important that the described associations are more often found at baseline than after one-year treatment. It is possible that when patients have a high inflammatory status, we find numerous associations between markers of bone and vascular status. After 12 months, the systemic inflammatory reactions may be under control, therefore, relationship between bone and vascular inflammatory processes may not be so evident. This proposal is further supported by results of the MANOVA analysis showing that disease activity or CRP at baseline influence the associations between bone and vascular biomarkers. All these observations also support that the one-year TNF inhibition indeed had significant effects on vascular-bone interactions.

Our results suggest that multiple associations may exist between molecular mechanisms of bone loss and atherosclerosis associated with arthritides. Furthermore, the baseline levels of some bone or vascular laboratory biomarkers may predict vascular or bone imaging markers after 6–12 months of anti-TNF treatment, respectively. Finally, the associations between bone and vascular status may be significantly affected by systemic inflammation and disease activity supporting the idea that arthritis enhances both CVD and OP already in early stage of the disease^1,25,39^.

As there has been only one report^31^, where vascular and bone status was assessed in arthritis, we could not compare our data with those reported by other groups. We^1^ and others^16–18,39^ have reviewed possible clinical, cellular and molecular interactions between the bone and vascular system both under non-inflammatory and inflammatory conditions. Moreover, as discussed above, primarily bone biomarkers, such as osteoclacin, CTX, osteoprotegerin, RANKL, sclerostin, DKK1, PTH, cathepsin K and vitamin D3, as well as essentially vascular and angiogenic markers, such as PDGFBB, angiopoietin 1 and 2, thrombospondin 1 and BNP have also been implicated in vascular and bone pathologies, respectively^1,7,9,10,16–18,20–25,29^. Regarding targeted therapies, several reports suggested that anti-TNF agents and other biologics may decrease arthritis-associated bone loss and suppress inflammatory atherosclerosis. Biologics may improve vascular and bone status including effects of biomarkers also assessed in the present study^1,4,6,8,11,32–38,40–42^.

In conclusion, to our best knowledge, this is the first study that simultaneously assessed vascular and bone status in a cohort of inflammatory arthritis patients treated with biologics. We assessed a high number of vascular and bone biomarkers in these patients to gain a better insight into their possible interactions and tried to find vascular and bone marker determinants of inflammatory bone loss and atherosclerosis, respectively. This study has some limitations. Due to the relatively low number of patients, we could not compare RA and AS patients or examine the effect of etanercept and certolizumab pegol separately. Further studies are needed to evaluate the potential determinants of CVD and OP and the relationship between bone and vascular pathology in inflammatory rheumatic diseases.

## Data Availability

All data are included in the manuscript in the tables.
